# It may cost an arm and a leg: workers value and occupational fatality rates in the U.S.

**DOI:** 10.1186/s12889-021-11117-9

**Published:** 2021-06-13

**Authors:** Leah S. Klos, Frank B. Giordano, Stacy A. Stoffregen, Miki C. Azuma, Jin Lee

**Affiliations:** Department of Psychological Sciences, Kansas State University, 1114 Mid-Campus Dr, KS 66506 Manhattan, USA

**Keywords:** Worker value, Minimum wage, Workers compensation, Occupational safety and health disparity

## Abstract

**Background:**

The present study aims to observe how societal indicators of workers’ values at the state-level are related to health and safety outcomes, particularly major injuries and fatalities in the U.S. Underscoring workforce flexibility and workability over workforce stability and safety might be indicative of the worth of workers which can be associated with occupational safety and health concerns.

**Methods:**

Linear regression analysis with a log-transformed dependent variable was adopted to examine how the state-level indicators of worker value in terms of 1) minimum wage, using data from 2015; 2) average of workers’ compensations for the loss of an arm, hand, leg, or foot in 2015 were concurrently and prospectively associated with occupational fatality rates averaged across 2015, 2016 and 2017. Socioeconomic contextual variables such as education level, GDP per capita, and population at the state-level were controlled for.

**Results:**

The present study showed that state-level quantitative indicators of how workers are valued at work, namely minimum wage and workers’ compensation benefits, were significantly and negatively associated with fatality rates in the following year.

**Conclusions:**

The present study illustrates the gap in how workers are valued across the U.S. The study speaks to the importance of contextual factors regarding worker value, as they can affect outcomes of health and safety culminating at a state-level.

**Supplementary Information:**

The online version contains supplementary material available at 10.1186/s12889-021-11117-9.

## Background

Workers’ value of their health and safety imposed by contextual factors might be associated with the amount of occupational effort and resources used to protect workers. In the context where workers are more valued, more attention will be given and greater quantity and quality of safety protections will be offered for their safety and health. Alternatively, where workers are less valued, they might suffer from greater workplace hazards and extreme outcomes such as occupational fatalities due to lesser safety protections.

A key motivation for not providing adequate safety education and training is to reduce organizational resources, saving time and money [1, 2]. Even while adhering to federal safety and health standards (e.g., improving safety environments, providing safety equipment), organizations may divert organizational resources away from safety and health practices in order to focus on positive business outcomes (e.g., increasing productivity, meeting deadlines). In fact, many organizations emphasize organizational success over matters of safety [3] such that workers can be exposed to hazards they were previously safeguarded against.

Sensemaking theory [4] contends that people rationalize and give meaning to their experiences based on pertinent contextual information. It helps explain how people develop a consensus on values and beliefs which can be used as plausible reasons for their behaviors in a given context. According to sensemaking theory, people develop shared perceptions on which behaviors and practices are acceptable or not acceptable throughout the ongoing process of retrospective assessment of social norms/standards and common practices.

Haas and Yorio [5] reframe the sensemaking model in terms of risk assessment and state that sensemaking can be viewed as a risk management process which allows everyone in the workplace to identify hazards, communicate risks, and respond accordingly. Sensemaking begins with an observation of organizational cues and workplace behaviors by employees. If any disparity is noted, employees engage in the enactment of addressing this disparity by aligning behaviors to the organizational cues or devaluing, overlooking, and annulling the cues. Behaviors that are reinforced or not penalized would be retained and referenced to as the base of an organization’s policies and procedures. This process applies to employers as well such that they can make sense of an adequate level of managerial standards in the promotion of occupational safety and health by observing societal norms and common practices of appraising workers’ value. As long as their current practices do not incur any notable backlash from governing authority and organizational members, policies and procedures of their organization would be maintained. In turn, organizations tend to reinforce and solidify their cultures through a series of attraction, selection, and attrition processes [6, 7].

The present study, on the continuum of the pilot study of Lee and Klos [8], posits the different standards and practices of workers’ value among the 50 United States and the federal District of Columbia (D.C.) can lead employers to have a certain sense as to the value of their workers, which will be referred to as worker value throughout the remainder of this study. Specifically, we focus on minimum wage and workers’ compensation benefits.

Among the many indicators of worker value, minimum wage was chosen because it is a set value and oftentimes the basis of the calculation of labor cost [9]. Also, all 50 states of the U.S. and D.C. have jurisdictional guidelines for minimum wages. A number of socioeconomic factors are associated with minimum wage. Low minimum wage might be indicative of affordability of labor as well as easiness to find substitutes when incumbent workers become unavailable or lose their workability. Moreover, low minimum wage itself has been noted as potential occupational safety and health hazards because of its impact on suboptimal access to quality medical care [10–12]. Additionally, minimum wage can reflect a state’s protection policies towards workers.

Another indicator of worker value considered in the present study is workers’ compensation benefits. Workers’ compensation is a form of insurance providing financial resources to cover lost wages, medical costs, and ongoing care expenses to injured workers during employment. The employers are the policy holders of workers’ compensation insurance. There is no federal oversight of workers’ compensation programs, which are regulated by each state [13]. The American Public Health Association [13] found that many employers believe the incidence rate of injury has plateaued or declined, implying that hazardous work conditions are no longer a meaningful threat to workers. To avoid increased premiums in a seemingly safer work environment, companies can misclassify workers and underreport payroll in an effort to obtain a lower premium [14]. Despite the workers’ compensation cuts and the consistently decreasing costs of workers’ compensation, self-insured employers argue that high workers’ compensation costs will not attract economic growth and in order to stay competitive, workers’ compensation benefits costs must stay relatively low [13, 14]. In sum, workers’ compensation is necessary to protect workers, but more adequate amounts of workers’ compensation benefits pose greater financial burden to employers. As a result, the amount of workers’ compensation benefits can imply the worker value over competing business demands.

Overall, people make decisions in consideration of contextual factors on acceptable and desirable behaviors [15]. Accordingly, local governments, organizations, and employers may interpret minimum wage and workers’ compensation benefits as specific contextual factors regarding worker value and use this information to leverage human resources to achieve industrial and economic progress. If workers are inadequately valued, it does not violate common sense to treat workers as expendable commodities [16].

### Hypotheses

The present study aimed to explore how minimum wage and workers’ compensation benefits in 2015 are associated with the average fatality rates across 50 United States and D.C. in 2015 through 2017. To this end, the following hypotheses were examined:

- *Hypothesis 1. Minimum wage is significantly and negatively associated with average fatality rates.*
- *Hypothesis 2. Average workers’ compensation benefits for major body part loss is significantly and negatively associated with average fatality rates.*

## Methods

The present study utilized archival data on five state-level variables (*n* = 51) categorized into three groups including key-study, contextual, and control variables. Key-study variables were 1) minimum wage in USD units ($), using data from 2015; 2) average of workers’ compensations for the loss of an arm, hand, leg, or foot in 2015.

### Minimum wage

We used the 2015 minimum wage data from the U.S. Department of Labor [17]. The 2015 minimum wage data had a range of $2.00 (Oklahoma) to $10.50 (D.C.) with a mean of $7.67 (SD = $1.34). For states with different minimum wage standards for large and small employers, we used the minimum wage standards that had the smallest value. In fact, a sizeable portion of workers are hired through small businesses in 2017, such as in Minnesota (47.8%), Montana (65.2%), Nevada (42.0%), Ohio (46.0%), and Oklahoma (52.8%), while 47.5% of the private workforce in the U.S. was employed by small businesses [18].

### Average of workers’ compensations for the major body part loss

The four body parts – arm, hand, leg, and foot – were chosen to be the focus for looking at average workers’ compensation for a few key reasons. These four body parts are major body parts that are commonly injured during work and the amount of workers’ compensation for the loss of other body parts is generally highly correlated with the workers’ compensation for the loss of the four chosen body parts (e.g., correlations with the workers’ compensation for the loss of eye ranged from .88 to .95). Also, data were available in all 50 states and D.C. for all four of these body parts (i.e., the amount of workers’ compensation for the loss of eye info was not available from 10 out of 50 states). The average of the workers’ compensation benefits for the loss of the four major body parts can be indicative of the overall amount of financial support from the employer for workers who are permanently disabled and lost workability due to their work.

**Fig. 1:**
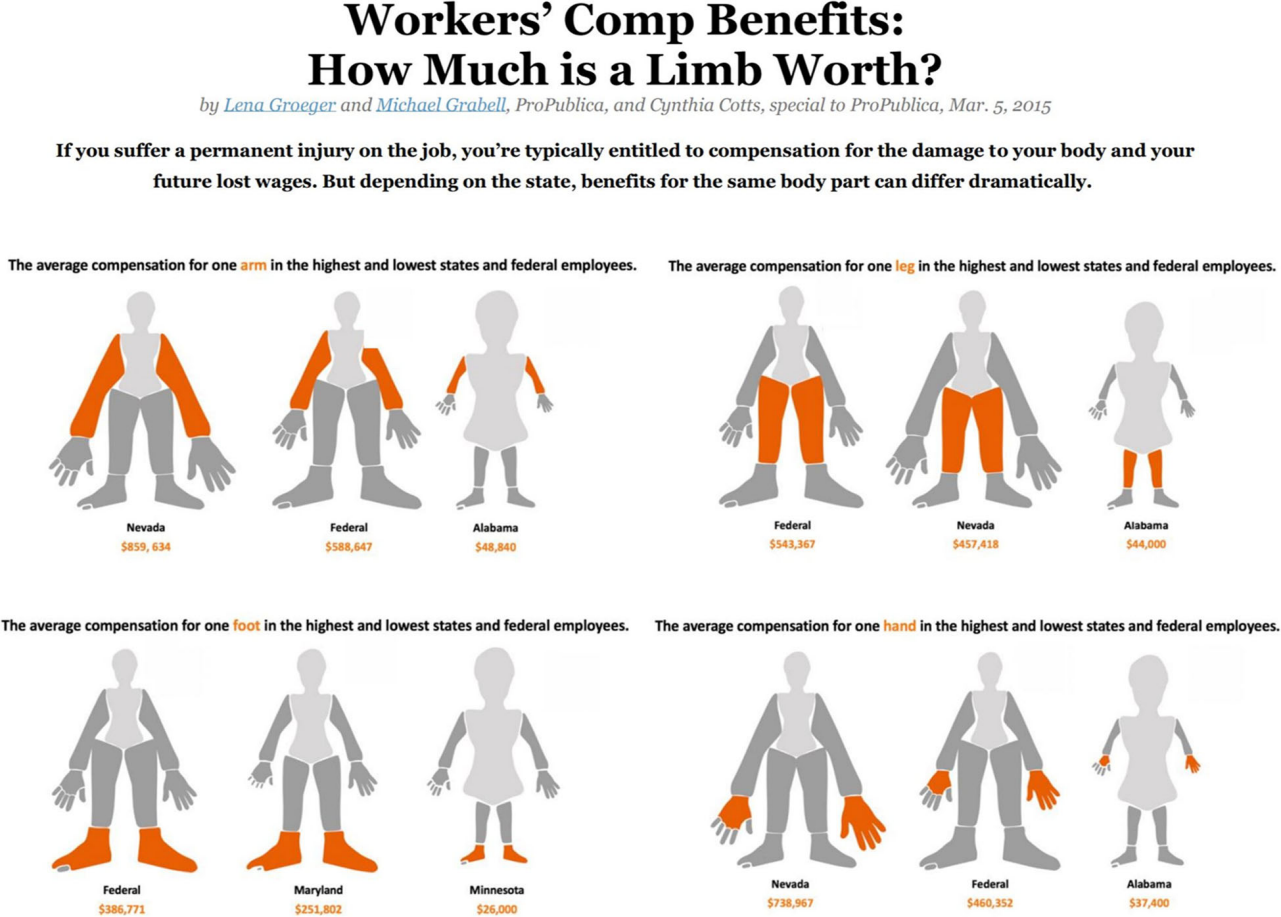
Visualization of Workers’ Compensation Benefits across States and Federal Employees by ProPublica. *Notes.* Image originally from [19] and has been adapted with permission. Permission was given on 11/19/2019 via email correspondence with the original authors. The source of the image can be found at https://projects.propublica.org/graphics/workers-compensation-benefits-by-limb

The data for all four body parts were obtained from a ProPublica article by Groeger, Grabell, and Cotts [19]. ProPublica calculated the maximum benefit injured workers can receive for the total loss or amputation of various parts by researching the law for all 50 states and D.C., following each state’s provided formula. The maximum benefit was determined by taking a 100% loss of each body part for a worker who earned enough to qualify for the state’s current maximum compensation rate. In cases where states assigned higher values for amputations, or if the injury occurred on a dominant hand, the highest value was used [20]. In this data, the average of workers’ compensation benefits for a permanently injured hand, arm, leg, or foot (for damage to body part and future lost wages) were $40,205 in Alabama and $568,027 in Nevada (national mean = $157,944, SD = $97,767) and for each state the maximum benefit was used. This is illustrated for each of the four individual body parts in Fig. 1.

### Contextual variables

**Fig. 2:**
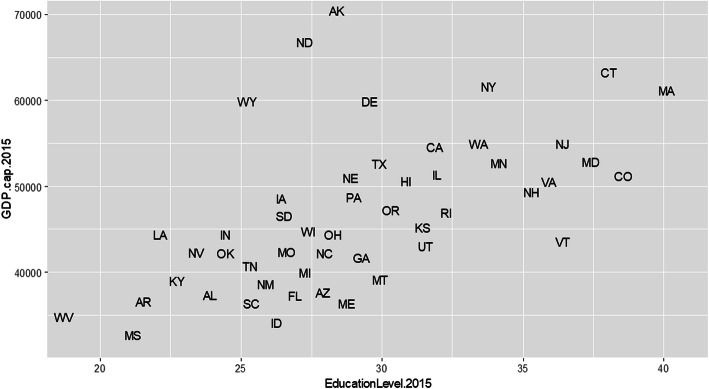
Education level (2015) and GDP per capita (2015) Across 50 States. *Notes.* GDP per capita in 2015 is in $USD unit; % of Completed College education (2015) suggests the percentage of people with bachelor’s degree or higher

Contextual variables were 1) education level per state defined by the % college degree earned in 2015 [21]; 2) GDP (gross domestic product) per capita in 2015 [22]. These variables were included because they can respectively serve as representatives of job type (i.e., more/less protected) [23] and regional economic development (see Fig. 2). A combination of both education and GDP per capita offers a useful snapshot of the primary industries in a particular state. For instance, Alaska has a low education level, but high GDP, which is reflected in its major industries being high risk with high return (e.g., natural resource development, fishing, and logging). Massachusetts has a high education level and GDP with its major industries being low risk with high return (e.g., healthcare, education, and finance). Mississippi has low education levels and GDP with its major industries being high risk with low return (e.g., agriculture and retail). Also, it is worth noting that minimum wage might be influenced by the standard of living in the state while it oftentimes is reflected by GDP per capita [24].

### Control variable

The control variable for the present study was the population for each state according to the national census data from 2010 [25]. This variable was included because it can serve as the surrogate for availability and cost of labor as suggested by the demand-supply model [26, 27].

A linear regression analysis was conducted to examine the prospective relationship between these five variables and the average occupational fatality rates for the years 2015 through 2017 [28], operationalized as the number of workers killed at work per 100,000 workers. The dependent variable, fatality rates, was log-transformed.. The analysis was conducted with archival data using the statistical software R.

## Results

**Fig. 3:**
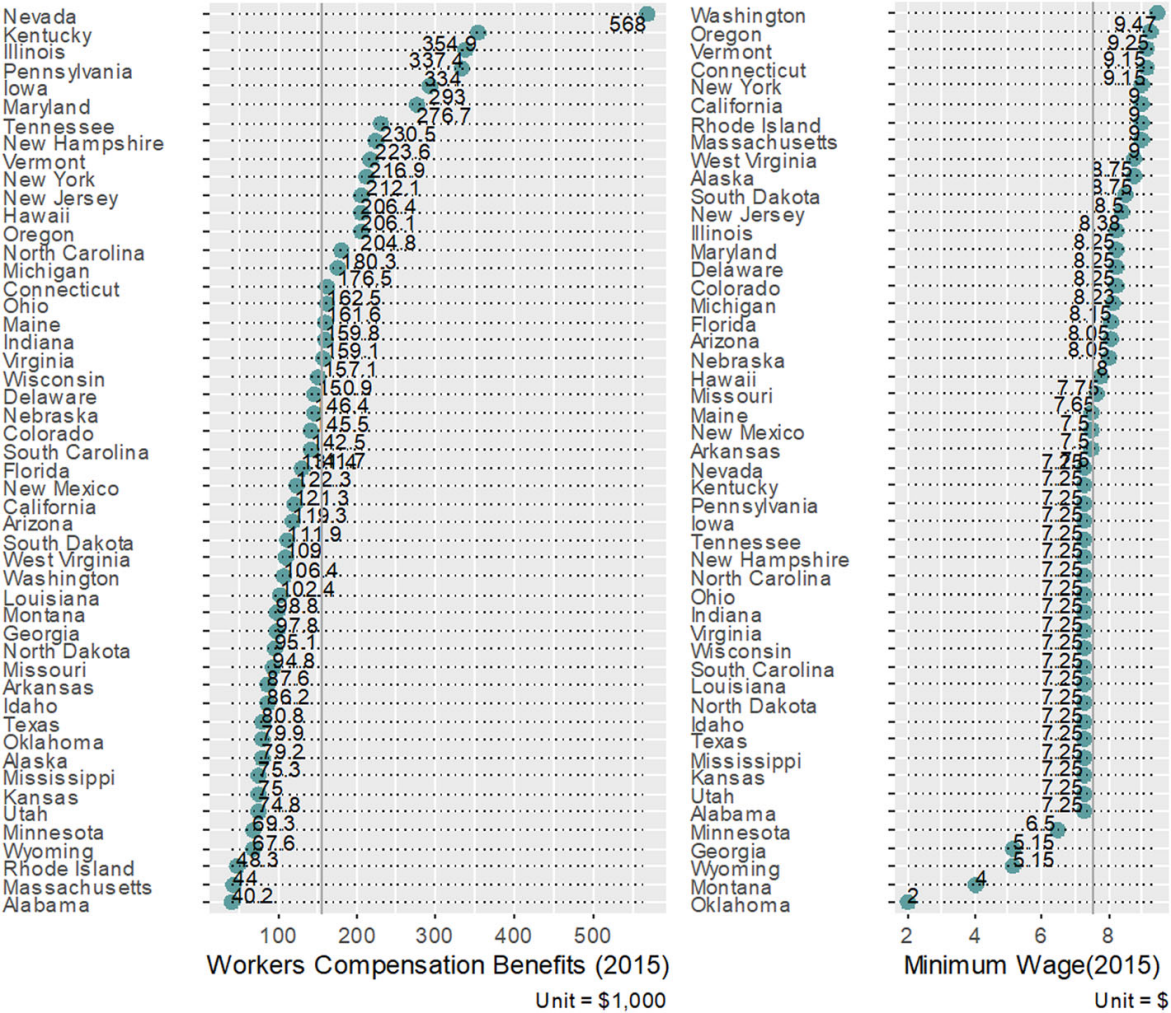
Average of Maximum Workers’ Compensation Benefits for the Loss of an Arm, Hand, Leg, or Foot and Minimum Hourly Wage in 2015 across 50 U.S. states. *Notes.* When a state has double standards for minimum wage depending on the size of business, we centered on the smaller amounts of minimum wage

**Fig. 4:**
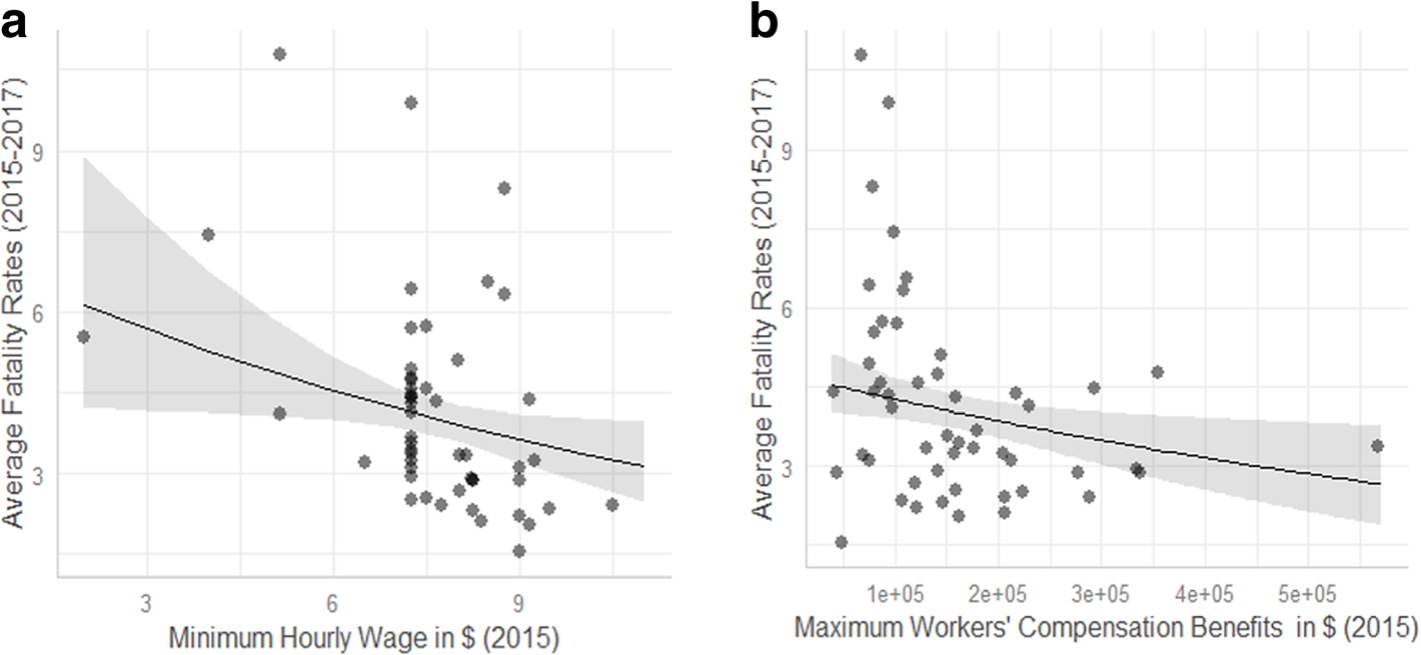
**A** Predicted Values of Fatality Rates based on the Relationship between Minimum Hourly Wage (2015) and Average Fatality Rates (2015–2017). *Notes.* The unstandardized fatality rate variable was used to create the current figure. The shaded region around the trend line suggests the 95% confidence interval. **B** Predicted Values of Fatality Rates based on the Relationship between Average Maximum Workers’ Compensation Benefits (2015) and Average Fatality Rates (2015–2017). *Notes.* The unstandardized fatality rate variable was used to create the current figure. The shaded region around the trend line suggests the 95% confidence interval

Descriptive statistics and correlations of the study variables are presented in an additional file [see Additional file 1]. As summarized in Table 1 and Fig. 3, both workers’ compensation benefits (B = − 0.09, SE = 0.04, *p* = 0.042) and minimum wage (B = − 0.11, SE = 0.05, *p* = 0.024) in 2015 were significantly and negatively associated with the average fatality rates in 2015 through 2017. The outcome variable of fatality rates was log-transformed, so the results from the linear regression analysis were exponentiated to allow for a more meaningful interpretation of the main variables of interest. A standard deviation increase in average workers’ compensation benefits was associated with an 8.3% reduction in fatality rates (95% CI: − 15.7% [= 1 – exp.(− 0.171)] to −.03% [= 1 – exp.(− 0.003]). A standard deviation increase in minimum wage was associated with a 10.3% reduction in fatality rates (95% CI: − 18.3% [= 1 – exp.(− 0.202)] to − 1.5% [= 1 – exp.(− 0.15)]). These findings supported hypotheses 1 and 2, as seen in Fig. 4A and B. Regarding the contextual and control variables, education level was negatively associated with fatality rates (B = − 0.33, SE = 0.06, *p* = 0.000) while GDP per capita was positively associated with fatality rates (B = 0.22, SE = 0.06, *p* = 0.001). Population for each state was negatively associated with fatality rates (B = − 0.07, SD = 0.04, *p* = 0.076).

**Table 1:** Hypotheses testing results based on a regression analysis

	DV = Log-Transformed Average Fatality Rates			
	B (SE)	*p*-value	Lower 95% CI	Upper 95% CI
(Intercept)	1.33 (.04)	0.000	1.25	1.42
*Study Variables*	− 0.09 (0.04)	0.042		
Average WCB in 2015			−0.17	− 0.00
Minimum Wage in 2015	−0.11 (0.05)	0.024	−0.20	− 0.02
*Contextual Variables*				
Education Level in 2015	−0.33 (0.06)	0.000	−0.46	− 0.21
GDP per capita in 2015	0.22 (0.06)	0.001	0.10	0.35
*Control Variables*				
Population in 2010	−0.07 (0.04)	0.076	−0.16	0.01

*Average WCB* $ Average of workers’ compensation benefits for the loss of an arm, hand, leg, or foot in 2015, *Minimum wage* $USD, data from 2015, *Education Level* % college degree earned in 2015, *GDP per capita* gross domestic product per capita in 2015, *Population* National census data from 2010, *DV* Log-transformed average fatality rates in 2015–2017, the number of workers killed at work per 100,000 workers

## Discussion

The present study showed that state-level quantitative indicators of worker value, namely minimum wage and workers’ compensation benefits, were significantly and negatively associated with the average fatality rate from 2015 to 2017. Within a culture of capitalism, in which profit can be prioritized over worker safety and workers can be inadequately valued, injured and diseased workers are more likely to be considered expendable [29]. Additionally, while workers’ compensation benefits are intended to protect workers, workers can still suffer from a loss of income due to decreased workability after incurring an occupational injury that warrants workers’ compensation [30]. Monetary informational and other regulatory indicators of worker value can serve as contextual factors that provide reference points for linking beliefs and ideas to broader networks of meaning which can augment both employers’ and employees’ sensemaking processes about occupational safety and health [15, 31]. These informational cues can be exacerbated considering that contracts are designed to efficiently defend against fraudulent claims by overinsuring small losses and underinsuring large injuries, thus restricting the monetary value that can be assigned to workers’ health [32]. Due to sensemaking, employers’ and employees’ project themselves onto the situation, which is often characterized by low minimum wage and insufficient workers’ compensation benefits. Subsequently, they observe the negative outcomes in their workplace such as improper safety leadership, training, and protections, and learn which organizational behaviors are acceptable [33].

Presumably, it is unrealistic to inflate minimum wage and workers’ compensation benefits arbitrarily in a short period of time. However, we can think about alternative protections for workers through social and organizational systems. For example, we may want to try to enhance the awareness of employers and employees regarding the inadequacy of the extant minimum wage and workers’ compensation benefits in the protection of workers. Raising awareness on workers’ compensation benefits is important as it has been shown that those who are in more precarious employment situations are not only more likely to get injured and need access to workers’ compensation benefits, they are less likely to be aware of workers’ compensation and the assistance it provides [34]. Additionally, many past studies have shown that a large number of workers do not file claims even when they are aware of these benefits and qualify for them [35–37]. Raising awareness could help workers receive needed protections such as workers’ compensation benefits.

Additionally, more prevention efforts can be made throughout the United States. There can be more collaborative efforts between organizations and government agencies to provide safety and health training/programs as well as access to occupational safety services (e.g., safety monitoring and hazard assessment). It has been shown that overtime, safety initiatives increase safety performance and reduce accidents, which in turn helps lower insurance costs for organizations [38]. Haley-Lock and Shah [39], describe that while employers are already minimally incentivized by public policy to participate in supportive employment practices, research has found that those who chose to participate in additional high involvement human resource management strategies helped reduce expenses related to turnover, hiring, and training. Furthermore, policy makers can focus on addressing the gaps in current policies and loopholes in implementation of these policies to mitigate the workplace safety and health related disparity between lower and higher income workers [40]. Additionally, public policy can put in place programs to incentivize employers to be more involved in the safety and health of their workers and provide resources to help these employers train and educate their workers on occupational safety.

In order to extend the present study conducted at the state-level, future research is needed to examine whether the minimum wage and workers’ compensation benefits at the state-level are indeed interpreted as worker value at the individual-level across the samples of employees and employers. Also, to address the limitation of the study findings based on archival data, a more controlled experimental approach utilizing a series of likely scenarios of judgment and decision making in terms of occupational safety and health investment as well as the provision of various financial support including salaries and workers’ compensation benefits can be adopted. Additionally, the present study utilized data on workers’ compensation benefits that did not differentiate between the two types of payouts for workers’ compensation benefits which are a lump sum or long-term payments over time. The present study utilized the maximum payment offered per injury, making the results a conservative estimate. Future research can investigate if there are differences in occupational safety and health outcomes based on these two different payout systems. Finally, the present study utilized three contextual variables, GDP, education level, and population, to control for the potential variation in common industry sectors and labor market situations across the 50 states and D.C. Alternative indicators more directly reflecting the characteristics of industries or workforce can be considered for future studies.

## Conclusions

Minimum wage and workers’ compensation benefits, which were chosen for the present study as the two state-level quantitative indicators of worker value, were significantly and negatively associated with fatality rates. The study speaks to the importance of contextual factors and their relationship with worker value, as they can be one of the many factors that affect outcomes of health and safety, culminating at a state-level. Further investigation into these contextual factors is needed to fully understand these relationships.

## Supplementary Information

**Additional file 1.**

## Data Availability

The dataset generated and/or analyzed during the current study are attached to the submission under supplemental materials. The data used is also publicly available and accessible at the following sources: U.S. Department of Labor [17], ProPublica [19], Bureau of Economic Research [21], Bureau of Economic Analysis [22], U.S. Census Bureau [25], and U.S. Bureau of Labor Statistics [28],

## Abbreviations

U.S: Unites States
D.C: District of Columbia
USD: United States dollar
GDP: Gross domestic product
WCB: Workers’ compensation benefits

## References

[1] Gun RT (1993). The role of regulations in the prevention of occupational injury. Saf Sci.

[2] Rousseau DM, Libuser C (1997). Contingent workers in high risk environments. Calif Manag Rev.

[3] Kartam NA, Flood I, Koushki P (2000). Construction safety in Kuwait: issues, procedures, problems, and recommendations. Saf Sci.

[4] Weick KE, Sutcliffe KM, Obstfeld D (2005). Organizing and the process of sensemaking. Organ Sci.

[5] Haas EJ, Yorio PL (2018). Using sensemaking theory to improve risk management and risk communication: what can WeLearn. Selected Issues Glob Health Commun.

[6] Schneider B (1987). The people make the place. Pers Psychol.

[7] Schneider B, Goldstein HW, Smith DB (1995). The ASA framework: an update. Pers Psychol.

[8] Lee J, Klos LS. Value on workers and occupational fatality rates in the U.S. Proceedings of the work, stress, and health. Philadelphia; 2019. p. 290-1. Available from: https://www.apa.org/wsh/past/2019/2019-program.pdf

[9] Rebitzer JB, Taylor LJ (1995). The consequences of minimum wage laws some new theoretical ideas. J Public Econ.

[10] Lenhart O (2017). Do higher minimum wages benefit health? Evidence from the UK. J Policy Anal Manage.

[11] Leigh JP, Du J (2018). Effects of minimum wages on population health. InHealth Policy Brief. Health Affairs.

[12] Leigh JP, Leigh WA, Du J (2019). Minimum wages and public health: a literature review. Prev Med.

[13] APHA (2017). The critical need to reform workers’ compensation.

[14] Grabell M, Berkes H (2015). The Demolition of Workers’ Comp.

[15] Brown AD, Stacey P, Nandhakumar J (2008). Making sense of sensemaking narratives. Hum Relat.

[16] Kaufman BE, Beaumont RA, Helfgott RB (2003). Industrial relations to human resources and beyond: the evolving process of employee relations management. ME Sharpe.

[17] U.S. Department of Labor (2020). Changes in Basic Minimum Wages in Non-Farm Employment Under State Law: Selected Years 1968 to 2019. U.S. Department of Labor.

[18] U.S. Small Business Administration (2018). 2018 Small Business Profiles for the States and Territories: The U.S. Small Business Administration.

[19] Groeger L, Grabell M, Cotts C (2015). Workers’ Comp Benefits: How Much is a Limb Worth?.

[20] Grabell M, Groeger L (2015). Methodology for Workers’ Comp Benefits: How Much is a Limb Worth?.

[21] Bureau of Economic Research (2020). Educational Attainment, Annual: Bachelor’s Degree or Higher by State.

[22] Bureau of Economic Analysis (2020). U.S. states by GDP per capita (chained 2009 dollars).

[23] Mainert J, Niepel C, Murphy KR, Greiff S (2019). The incremental contribution of complex problem-solving skills to the prediction of job level, job complexity, and salary. J Bus Psychol.

[24] Ringen S (1991). Households, standard of living, and inequality. Rev Income Wealth.

[25] U.S. Census Bureau, Population Division (2015). Annual Estimates of the Resident Population: April 1, 2010 to July 1, 2015.

[26] Kaufman BE (2009). Promoting labour market efficiency and fairness through a legal minimum wage: the Webbs and the social cost of labour. Br J Ind Relat.

[27] Webb S (1912). The economic theory of a legal minimum wage. J Polit Econ.

[28] U.S. Bureau of Labor Statistics (2018). Census of Fatal Occupational Injuries (CFOI) - Current and Revised Data.

[29] Mogensen V (2008). Worker safety under siege: labor, capital, and the politics of workplace safety in a deregulated world. ILR Rev.

[30] Powell D, Seabury S (2018). Medical care spending and labor market outcomes: evidence from workers’ compensation reforms. Am Econ Rev.

[31] Weick KE (1995). Sensemaking in organizations.

[32] Crocker KJ, Morgan J (1998). Is honesty the best policy? Curtailing insurance fraud through optimal incentive contracts. J Polit Econ.

[33] Thurlow A, Mills JH. Change, talk and sensemaking. J Organ Chang Manag. 2009;22(5):459–79.

[34] Quinlan M, Mayhew C (1999). Precarious employment and workers’ compensation. Int J Law Psychiatry.

[35] Biddle J, Roberts K (2003). Claiming behavior in workers’ compensation. J Risk Insur.

[36] Probst TM, Brubaker TL, Barsotti A (2008). Organizational injury rate underreporting: the moderating effect of organizational safety climate. J Appl Psychol.

[37] Shannon HS, Lowe GS (2002). How many injured workers do not file claims for workers’ compensation benefits?. Am J Ind Med.

[38] Hoonakker P, Loushine T, Carayon P, Kallman J, Kapp A, Smith MJ (2005). The effect of safety initiatives on safety performance: a longitudinal study. Appl Ergon.

[39] Haley-Lock A, Shah MF (2007). Protecting vulnerable workers: how public policy and private employers shape the contemporary low-wage work experience. Fam Soc.

[40] Baron SL, Beard S, Davis LK, Delp L, Forst L, Kidd-Taylor A, Liebman AK, Linnan L, Punnett L, Welch LS (2014). Promoting integrated approaches to reducing health inequities among low-income workers: applying a social ecological framework. Am J Ind Med.

