# Supplementary material for: It may cost an arm and a leg: workers value and occupational fatality rates in the U.S
Source: BMC Public Health. 2021 Jun 13;21:1133. doi: 10.1186/s12889-021-11117-9 (PMC8201718; doi:10.1186/s12889-021-11117-9)
Supplement: Supplementary file 1 — Additional file 1. [file 12889_2021_11117_MOESM1_ESM.docx]

| Table 1. Descriptive statistics and correlations among the study variables. | | | | | | | | |
| --- | --- | --- | --- | --- | --- | --- | --- | --- |
|  | Mean (SD) | 1 | 2 | 3 | 4 | 5 | 6 |  |
| 1. Average WCB | 172K (112K) | - |  |  |  |  |  |  |
| 2. Minimum Wage | 7.57 (1.32) | .12 | - |  |  |  |  |  |
| 3. Education Level | 29.08 (4.92) | .01 | .35* | - |  |  |  |  |
| 4. GDP per capita | 47106.70 (8934.92) | -.01 | .27 | .58** | - |  |  |  |
| 5. Population | 6163K (6848K) | .05 | .16 | .15 | .12 | - |  |  |
| 6. Average Fatality Rates | 4.19 (1.93) | -.29* | -.41** | -.50** | .05 | -.33* | - |  |
| *Notes*. ** p < .01; * p <.05; Average WCB = $ Average of workers’ compensation benefits for the loss of an arm, hand, leg, or foot in 2015; Minimum wage = $, data from 2015; Education Level = % college degree earned in 2015; GDP per capita = gross domestic product per capita in 2015; Income Gap = Gini index from the national data from 2010 (greater value indicates greater income gap); Population = National census data from 2010; Average Fatality Rates = Average fatality rates in 2015 -2017, the number of workers killed at work per 100,000 workers. | | | | | | | | |
